# Comparison of Size of Pulmonary Artery and Its Branches on Transthoracic Echocardiography Versus Computed Tomographic Angiography in Patients with Tetralogy of Fallot

**DOI:** 10.7759/cureus.9060

**Published:** 2020-07-08

**Authors:** Shah Nawaz Sathio, Abdul S Shaikh, Hussain Korejo, Veena Kumari, Naresh Kumar, Arshad Sohail, Mujeeb U Rehman, Najma Patel

**Affiliations:** 1 Paediatric Cardiology, National Institute of Cardiovascular Diseases, Karachi, PAK; 2 Paediatric Cardiology, Rehman Medical Institute, Peshawar, PAK

**Keywords:** pulmonary stenosis, tetralogy of fallot, transthoracic echocardiography, computed tomographic angiography

## Abstract

Background

Transthoracic echocardiography (TTE) plays a vital role in the assessment of the surgical management of patients with tetralogy of Fallot (TOF). Accurate assessment of the main pulmonary valve annulus, main pulmonary artery (MPA), and branch pulmonary arteries are crucial for decision-making regarding the surgical approach in the form of total correction. It is also important for performing a systemic-to-pulmonary artery shunt operation and affects the outcome. In some patients with poor echogenic windows, it is sometimes difficult to obtain accurate measurements. Cardiac computed tomographic angiography (CTA) can be a superior diagnostic modality. Therefore, the aim of this study was to evaluate the degree of agreement between TTE and CTA in assessing the main pulmonary valve annulus and the size of the MPA and its branches among patients with TOF patients.

Methodology

Patients above one year of age, with TOF, presented during the study period of six months - from January 1, 2019, to June 30, 2019, were included in the study. All the patients had TTE and cardiac CTA to assess the annulus and the size of the MPA and its branches (right pulmonary artery (RPA) and left pulmonary artery (LPA)). CTA measurement of all parameters was compared with TTE measurement of the same on three different views each by computing the Bland-Altman plot and Pearson correlation coefficients.

Results

A total of 73 TOF patients were included in this study. The correlation coefficients between CTA and TTE for the measurement of the annulus were 0.767 and 0.833 for the parasternal short-axis view and the subcostal view, respectively. The correlation coefficients between CTA and TTE for the measurement of MPA were 0.820 and 0.866 for the parasternal short-axis view and the suprasternal view, respectively. The correlation coefficients between CTA and TTE for the measurement of RPA were 0.883 and 0.897 for the parasternal short-axis view and the suprasternal view, respectively. Similarly, the correlation coefficients between CTA and TTE for the measurement of LPA were 0.848 and 0.877 for the parasternal short-axis view and the suprasternal view, respectively.

Conclusion

In conclusion, there is a strong correlation and agreement between cardiac CTA and TTE for the assessment of the annulus and the size of the pulmonary artery (PA) and its branches in patients with TOF.

## Introduction

Tetralogy of Fallot (TOF) has a variable and complex anatomy, especially of the pulmonary arteries (PAs). Surgical options and approaches depend a lot on the size of the pulmonary vessels and annulus. Therefore, an organized imaging approach, including multiple modalities, may be required in certain patients. Several diagnostic tools can be used, either alone or in combination, for imaging purposes in TOF, depending on the indications, patient’s age and clinical condition, availability and local expertise, the cost of each tool, as well as the possible need for intervention.

Besides the other information like additional ventricular septal defect (VSD) additional lesions, ventricular function, the severity of right ventricular (RV) obstruction, coronary artery anomalies, aortopulmonary collaterals, and the size of the pulmonary annulus and arteries are crucial in deciding the type of surgery and surgical approach. Like a hypoplastic annulus needs augmentation, hypoplastic pulmonary arteries may need augmentation or, if very small, total correction may not be feasible. Similarly, discrete stenotic lesions may need to be addressed either preoperatively or postoperatively. Even systemic pulmonary shunts need a proper assessment of pulmonary artery sizes.

Transthoracic echocardiography (TTE) alone is adequate for achieving the objective of imaging in average TOF patients for planning surgical repair [1]. Cardiac computed tomographic angiography (CTA) should be used as a reserved tool for specific indications like hypoplastic pulmonary arteries, coronary anomalies crossing the right ventricular outflow tract (RVOT), and aortopulmonary collaterals [2].

The surgical management of TOF depends upon the detailed anatomy of pulmonary arteries, along with the severity and type of pulmonary obstruction [3]. TTE plays a vital role in the detection and assessment of site, size, and severity of pulmonary stenosis [4], but due to the retrosternal location of the right ventricular outflow tract (RVOT) and the pulmonic valve, their accurate assessment may be difficult. Therefore, cardiac CTA can be a superior diagnostic modality, as it can provide valuable information on the anatomy of the PA tree and RVOT [5].

In this study, our aim was to compare the assessment of the annulus and the size of the PA and its branches on TTE and CTA among the patients presented with TOF at a tertiary care cardiac center.

## Materials and methods

This study was carried out at the department of pediatric cardiology at the National Institute of Cardiovascular Diseases (NICVD), Karachi, after approval from the institutional ethical review committee (ERC-02/2019). Patients above one year of age with tetralogy of Fallot (TOF) presented during the study period of six months - from January 1, 2019, to June 30, 2019, were included in the study. All patients were enrolled after taking informed consent from parents or guardians.

Echocardiography was performed in all the patients with TOF by a pediatric cardiologist before surgical referral. The diagnosis was confirmed with echocardiographic evidence of ventricular septal defect, right ventricular outflow tract obstruction (RVOTO), right ventricular hypertrophy, and overriding of the aorta.

TTE and CTA were performed in all of the patients. TTE investigations were done using a 3.0 to 5.0 MHz transducer with the Aplio i600 (Canon Medical Systems, Tochigi, Japan) and Toshiba Xario 200 (Toshiba Medical Systems, Otawara, Japa) ultrasound systems. Echocardiographic images included the parasternal short axis, suprasternal, and subcostal views. Cardiac CTA was done using a 64-multidetector computed tomography (MDCT) scanner (Aquilion, Toshiba Medical Systems) and dual-source CT (Siemens Medical Solutions, Forchheim, Germany).

TTE and CTA measurements of the annulus, main pulmonary artery (MPA), right pulmonary artery (RPA), and left pulmonary artery (LPA) were obtained. TTE assessment of all four parameters was obtained on the parasternal short-axis view. The assessment of MPA, RPA, and LPA was also obtained on TTE suprasternal view, and assessment of the annulus was obtained on the TTE subcostal view.

Collected data were analyzed using IBM SPSS version 21.0 (IBM Corp., Armonk, New York) and Microsoft Excel 2010 (Microsoft Corporation, Redmond, Washington), and mean ± standard deviation (SD), minimum, and maximum were calculated for the CTA and TTE measurement of the pulmonary annulus and MPA and its branches, the right pulmonary artery (RPA) and left pulmonary artery (LPA). CTA measurements of all parameters were compared with the TTE measurement of the same on three different views, each by computing a Bland-Altman plot. Average mean difference and 95% confidence interval of the mean differences as limits of agreement (LOA) were calculated by using the Bland-Altman plot. Pearson correlation coefficients were also computed. A two-sided tailed p-value ≥ 0.05 was taken as the level of significance.

## Results

CTA and TTE measurements of the annulus of the pulmonary artery (PA) and its branches were obtained for a total of 73 patients. The mean age of the patients was 8.3 ± 5.74 years, with 39.7% of patients of age less than five years, and 31.5% of patients between 11 and 18 years of age. Male patients were 67.1%, with the male to female ratio being 2:1. The gender and age distribution of the patients are provided in Table 1.

**Table 1:** Gender and age distribution of the patients SD = standard deviation

Characteristics	Total
Total (N)	73
Gender	
Male	67.1% (49)
Female	32.9% (24)
Age (years)	
Mean ± SD	8.3 ± 5.74 years
≤ 5 years	39.7% (29)
6 to 10 years	31.5% (23)
11 to 18 years	26% (19)
19 to 35 years	2.7% (2)

CTA measurements of the annulus, MPA, RPA, and LPA were 9.05 (±3.82 mm (SD), with the range 18.0-3.0 mm), 13.47 (±4.98 mm (SD), with the range 27.0-5.6 mm), 10.65 (±6.3 mm (SD), with the range 55-2.8 mm), and 12.46 (±6.16 mm (SD), with the range 39-2.8 mm), respectively. TTE and CTA measurements of the annulus, MPA, RPA, and LPA are presented in Table 2.

**Table 2:** Transthoracic echocardiography (TTE) and computed tomographic angiography (CTA) measurements of annulus, MPA, RPA, and LPA MPA = main pulmonary artery; RPA = right pulmonary artery; LPA = left pulmonary artery

Parameters	Mean ± Standard Deviation	Minimum - Maximum
Transthoracic echocardiography (TTE) - Parasternal short-axis view		
Annulus (mm)	9.17 ± 3.01	3.7 - 15.7
MPA (mm)	11.99 ± 5.06	6.3 - 36
RPA (mm)	9.08 ± 3.63	4.2 - 30.8
LPA (mm)	9.17 ± 4.76	3 - 31
Transthoracic echocardiography (TTE) - Suprasternal view		
MPA (mm)	11.58 ± 4.35	5.9 - 28
RPA (mm)	9.04 ± 3.37	4.3 - 28.7
LPA (mm)	9.06 ± 4.37	2.6 - 29.7
Transthoracic echocardiography (TTE) - Subcostal view		
Annulus (mm)	9 ± 3.06	3.5 - 16.1
Computed tomographic angiography (CTA)		
Annulus (mm)	9.05 ± 3.82	3 - 18
MPA (mm)	13.47 ± 4.98	5.6 - 27
RPA (mm)	10.65 ± 6.3	2.8 - 55
LPA (mm)	12.46 ± 6.16	2.8 - 39

The correlation coefficients between CTA and TTE for the measurement of the annulus were 0.767 and 0.833 for the parasternal short-axis and subcostal views, respectively. The correlation coefficients between CTA and TTE for the measurement of MPA were 0.820 and 0.866 for the parasternal short-axis and suprasternal views, respectively. The correlation coefficients between CTA and TTE for the measurement of RPA were 0.883 and 0.897 for the parasternal short-axis view and the suprasternal view, respectively. Similarly, the correlation coefficients between CTA and TTE for the measurement of LPA were 0.848 and 0.877 for the parasternal short-axis view and the suprasternal view, respectively.

**Figure 1:**
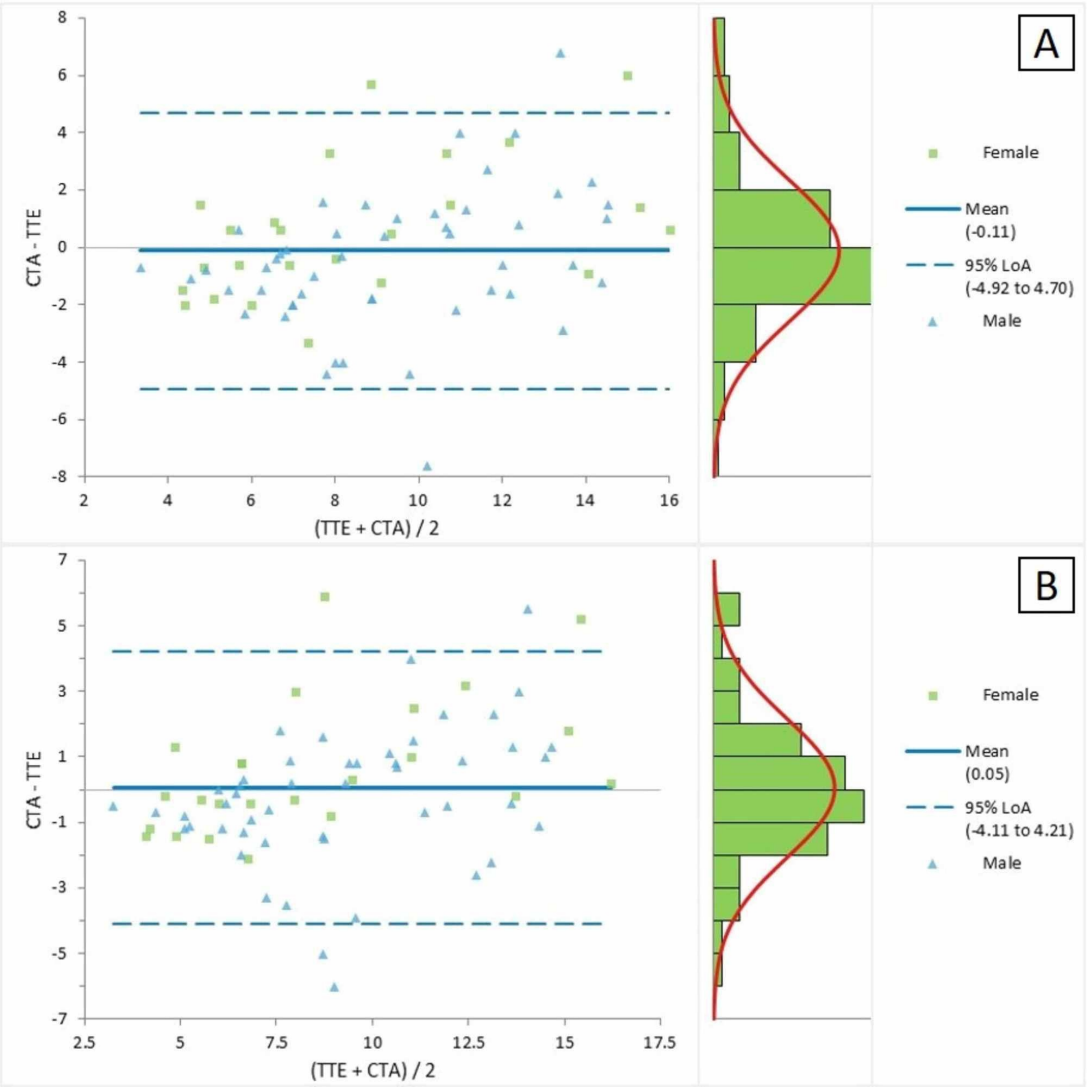
Bland-Altman plot for CTA and TTE (a) parasternal short axis and (b) subcostal views for the measurement of the annulus CTA = computed tomographic angiography; TTE = transthoracic echocardiography; LOA = limits of agreement

Figure 1A represents the Bland-Altman plot for the comparison of the CTA and TTE parasternal short-axis views for the measurement of the annulus. The average mean difference between the two measurements was -0.11, with LOA ranging between -4.92 and 4.70. The Bland-Altman plot in Figure 1B presented a comparison of the CTA and TTE subcostal views for the measurement of MPA; the mean difference between the two measures was detected as 0.05, with LOA ranging between -4.11 and 4.21.

**Figure 2:**
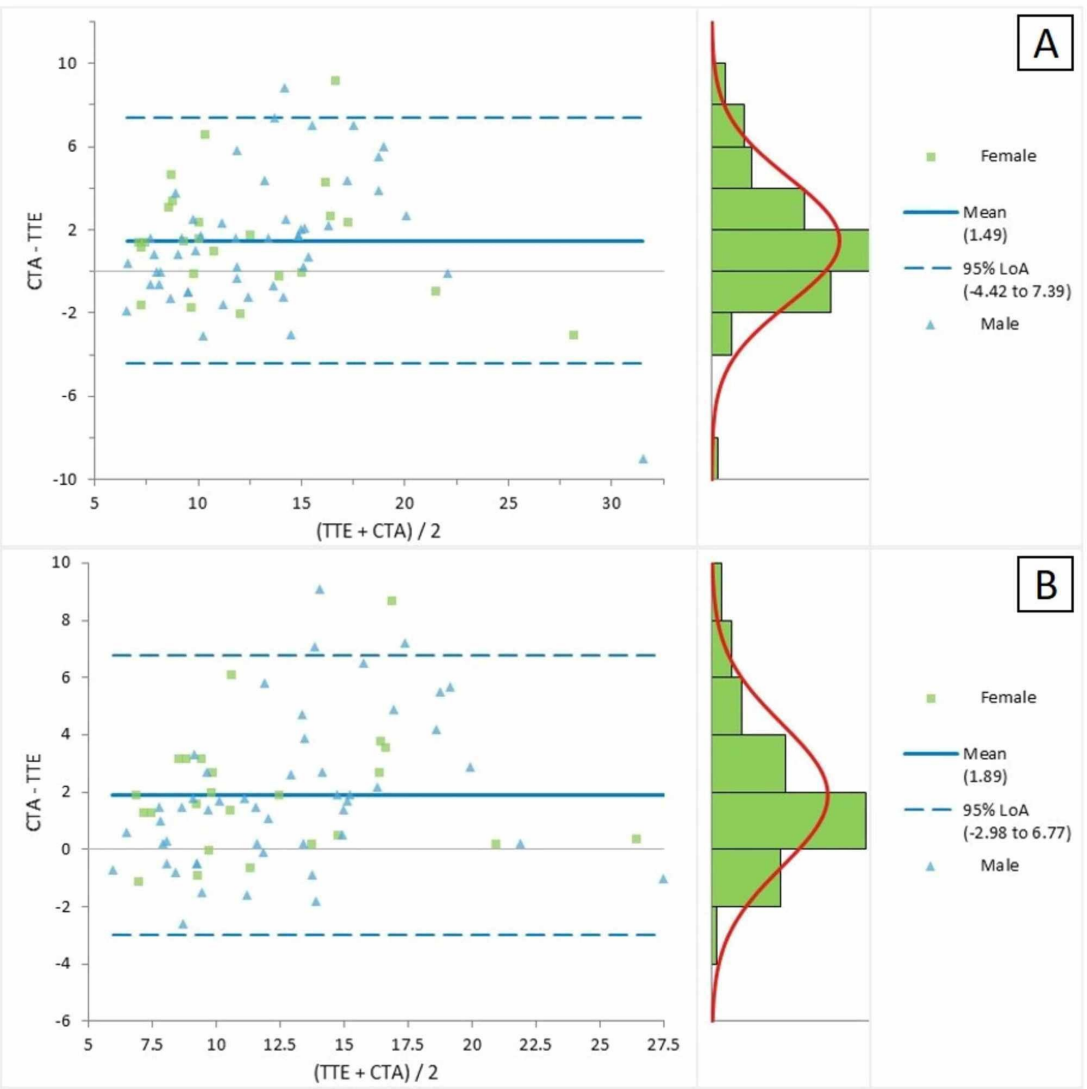
Bland-Altman plot for CTA and TTE (a) parasternal short axis and (b) suprasternal view for the measurement of the main pulmonary artery CTA = computed tomographic angiography; TTE = transthoracic echocardiography; LOA = limits of agreement

Figure 2A represents the Bland-Altman plot for the comparison of the CTA and TTE parasternal short-axis views for the measurement of MPA. The average mean difference between the two measurements was 1.49, with LOA ranging between -4.42 and 7.39. The Bland-Altman plot in Figure 2B presented a comparison of the CTA and TTE suprasternal views for the measurement of MPA; the mean difference between the two measures was detected as 1.89, with LOA ranging between -2.98 and 6.77.

**Figure 3:**
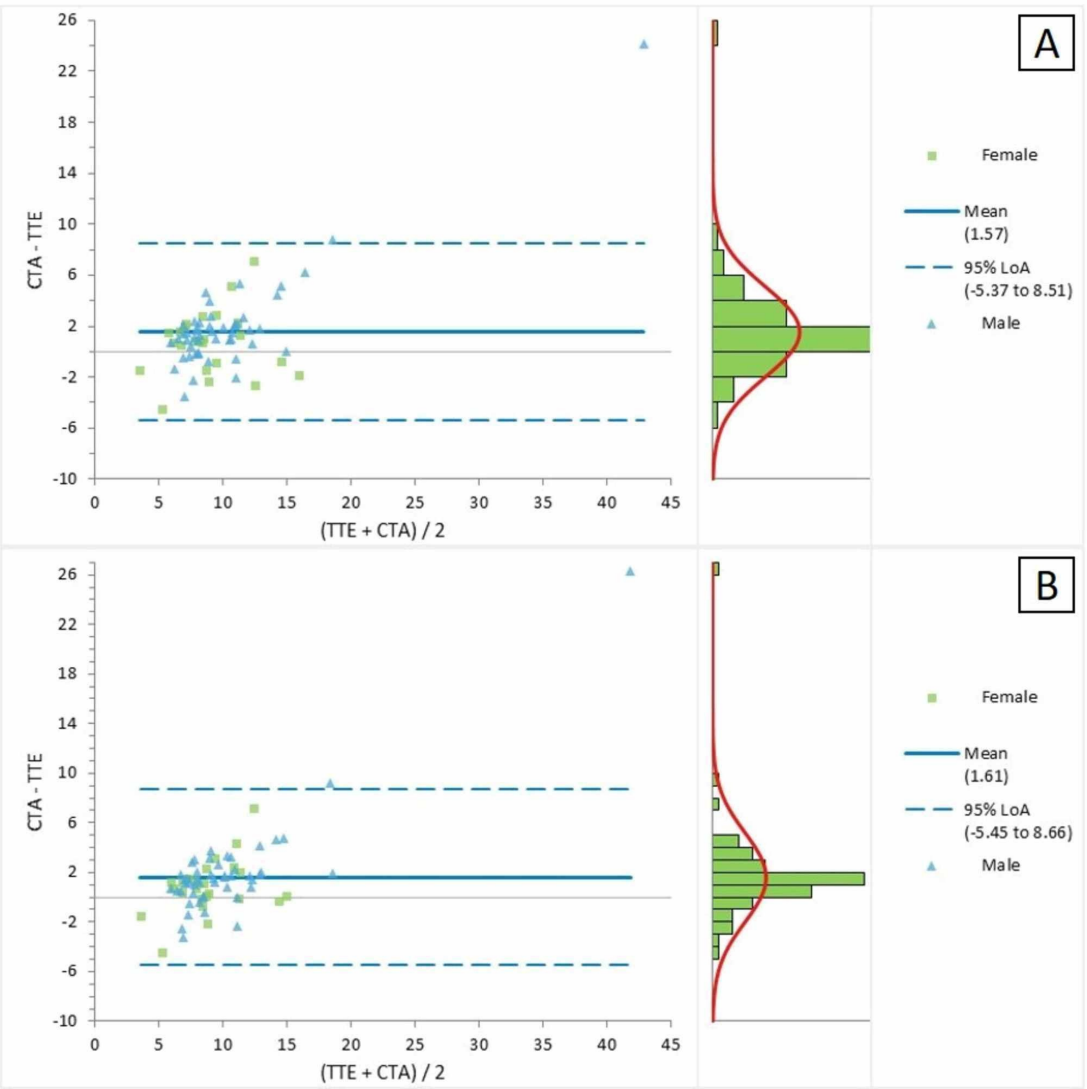
Bland-Altman plot for the CTA and TTE (a) parasternal short-axis and (b) suprasternal views for the measurement of the right pulmonary artery CTA = computed tomographic angiography; TTE = transthoracic echocardiography; LOA = limits of agreement

Figure 3A represents the Bland-Altman plot for the comparison of the CTA and TTE parasternal short-axis views for the measurement of RPA. The average mean difference between the two measurements was 1.57, with LOA ranging between -5.37 and 8.51. The Bland-Altman plot in Figure 3B presented a comparison of the CTA and TTE suprasternal views for the measurement of RPA; the mean difference between the two measures was detected as 1.61, with LOA ranging between -5.45 and 8.66.

**Figure 4:**
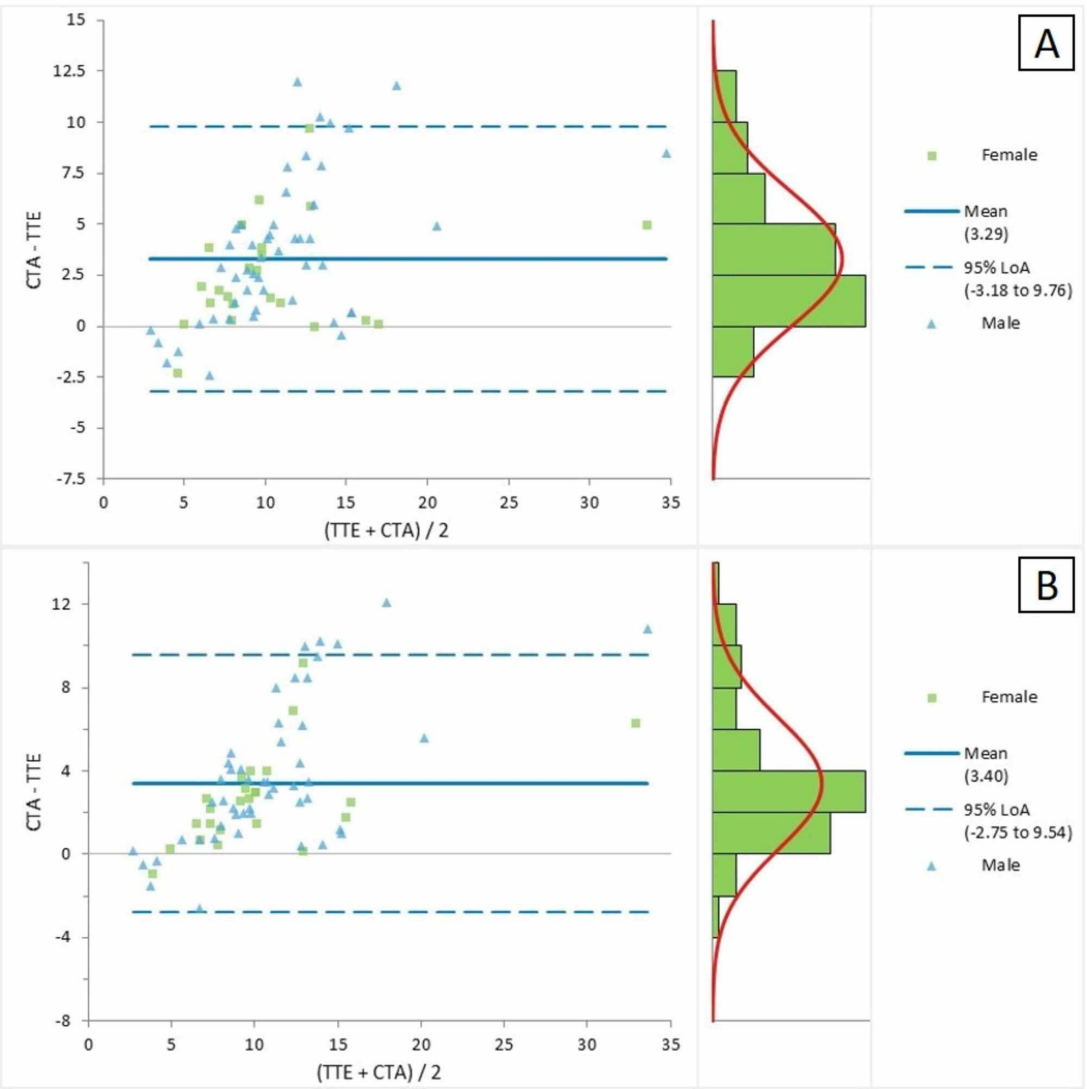
Bland-Altman plot for the CTA and TTE (a) parasternal short-axis and (b) suprasternal views for the measurement of the left pulmonary artery CTA = computed tomographic angiography; TTE = transthoracic echocardiography; LOA = limits of agreement

Figure 4A represents the Bland-Altman plot for the comparison of the CTA and TTE parasternal short-axis views for the measurement of LPA. The average mean difference between the two measurements was 3.29, with LOA ranging between -3.18 and 9.76. The Bland-Altman plot in Figure 4B presented a comparison of the CTA and TTE suprasternal views for the measurement of LPA; the mean difference between the two measures was detected as 3.40, with LOA ranging between -2.75 and 9.54.

## Discussion

TTE is the standard tool but due findings are limited at the time due to the anatomy and location of the RVOT. Therefore, this study was conducted with the aim to see the accuracy of the TTE measurement of the annulus and the size of the pulmonary artery and its branches by comparing these measurements with CTA.

CTA measurements were found to have a strong correlation and agreement with the corresponding TTE measurements. The CTA measurement of the annulus was found to be more strongly correlated with TTE measurements on the sub-costal view as compared to the parasternal short-axis view with correlation coefficients of 0.833 and 0.767, respectively. Stronger agreement between the TTE sub-costal view and CTA are evident from Figure 1B. Similarly, CTA measurements of MPA, RPA, and LPA were comparatively more strongly correlated with the TTE suprasternal view as compared to the TTE parasternal short-axis view with a correlation coefficient of 0.866 vs. 0.82, 0.897 vs. 0.883, and 0.877 vs. 0.848, respectively.

The use of CTA is on the rise, and it is becoming the preferred non-invasive tool for the more precise assessment of the main pulmonary artery and ascending aorta in patients with respiratory symptoms [6]. PA stenosis is a common finding in as high as 80% of TOF patients and presented with varying intensity of cyanosis [7-8]. An in-depth assessment of the anatomy of the pulmonary vasculature along with other cardiac defects for the better management and correction of TOF [7]. Angiographic studies are proved to be more accurate and precise than echocardiographic studies for the assessment of pulmonary vasculature [9-11]. The average diameter of the MPA, RPA, and LPA in healthy children on chest computed tomography (CT) scan was reported to be 19 mm, 12.1 mm, and 12.2 mm, respectively [6]. CTA measurements of the MPA and RPA in TOF patients in our study were comparatively smaller (13.47 ± 4.98 mm and 10.65 ± 6.3 mm) as compared to the reported diameter in normal children and LPA measurements were slightly higher in our study (12.46 ± 6.16 mm). Malik AA et al. reported CTA as better and more sensitive in detecting extracardiac findings of congenital heart disease, along with better delineation of anatomy [12].

TTE alone is adequate for achieving the objectives of the surgical management of patients with TOF. In the case of TOF with hypoplastic pulmonary arteries or annulus, CTA should be used as a reserved tool for the accurate assessment of MPA, annulus, and branch pulmonary arteries for decision-making regarding the surgical approach in the form of total correction or shunt operation and its outcome. Despite its limitation of small sample size, single-center experience, and the fact that surgical findings are not compared, this study provides useful information on the comparative advantage of CTA for the assessment of the PA and its branches.

## Conclusions

In conclusion, there is a strong correlation and agreement between cardiac CTA and TTE for the assessment of the annulus and the size of the pulmonary artery and its branches. TTE is quite reliable in patients with tetralogy of Fallot (TOF), having good echogenic windows.
